# PRMT5 is a therapeutic target in choroidal neovascularization

**DOI:** 10.1038/s41598-023-28215-w

**Published:** 2023-01-31

**Authors:** Anbukkarasi Muniyandi, Matthew Martin, Kamakshi Sishtla, Aishat Motolani, Mengyao Sun, Nathan R. Jensen, Xiaoping Qi, Michael E. Boulton, Lakshmi Prabhu, Tao Lu, Timothy W. Corson

**Affiliations:** 1 Department of Ophthalmology, Eugene and Marilyn Glick Eye Institute, Indiana University School of Medicine, Indianapolis, IN 46202 USA; 2 Department of Pharmacology & Toxicology, Indiana University School of Medicine, Indianapolis, IN 46202 USA; 3 Department of Ophthalmology and Visual Sciences, University of Alabama at Birmingham, Birmingham, AL 35233 USA; 4 Department of Biochemistry & Molecular Biology, Indiana University School of Medicine, Indianapolis, IN 46202 USA; 5 Department of Medical & Molecular Genetics, Indiana University School of Medicine, Indianapolis, IN 46202 USA

**Keywords:** Pharmacology, Macular degeneration, Molecular medicine

## Abstract

Ocular neovascular diseases including neovascular age-related macular degeneration (nvAMD) are widespread causes of blindness. Patients’ non-responsiveness to currently used biologics that target vascular endothelial growth factor (VEGF) poses an unmet need for novel therapies. Here, we identify protein arginine methyltransferase 5 (PRMT5) as a novel therapeutic target for nvAMD. PRMT5 is a well-known epigenetic enzyme. We previously showed that PRMT5 methylates and activates a proangiogenic and proinflammatory transcription factor, the nuclear factor kappa B (NF-κB), which has a master role in tumor progression, notably in pancreatic ductal adenocarcinoma and colorectal cancer. We identified a potent and specific small molecule inhibitor of PRMT5, PR5-LL-CM01, that dampens the methylation and activation of NF-κB. Here for the first time, we assessed the antiangiogenic activity of PR5-LL-CM01 in ocular cells. Immunostaining of human nvAMD sections revealed that PRMT5 is highly expressed in the retinal pigment epithelium (RPE)/choroid where neovascularization occurs, while mouse eyes with laser induced choroidal neovascularization (L-CNV) showed PRMT5 is overexpressed in the retinal ganglion cell layer and in the RPE/choroid. Importantly, inhibition of PRMT5 by PR5-LL-CM01 or shRNA knockdown of PRMT5 in human retinal endothelial cells (HRECs) and induced pluripotent stem cell (iPSC)-derived choroidal endothelial cells (iCEC2) reduced NF-κB activity and the expression of its target genes, such as tumor necrosis factor α (TNF-α) and VEGF-A. In addition to inhibiting angiogenic properties of proliferation and tube formation, PR5-LL-CM01 blocked cell cycle progression at G_1_/S-phase in a dose-dependent manner in these cells. Thus, we provide the first evidence that inhibition of PRMT5 impedes angiogenesis in ocular endothelial cells, suggesting PRMT5 as a potential therapeutic target to ameliorate ocular neovascularization.

## Introduction

Neovascular age-related macular degeneration (nvAMD) is a major cause of blindness, affecting an estimated 20 million older adults worldwide. It is characterized by macular neovascularization (MNV), the abnormal growth of new blood vessels (angiogenesis) from the choriocapillaris into the sub-retinal pigment epithelium (RPE) and/or subretinal space. nvAMD causes progressive degeneration of the neuroretina and RPE-choroid complex, leading to central vision loss^1^. Angiogenesis and inflammation are the hallmarks of nvAMD, where angiogenesis triggers endothelial cell proliferation and migration along with dysregulation of vascular endothelial growth factor (VEGF) as a key pathological factor^2^. Ocular neovascularization also underlies other blinding eye diseases, such as proliferative diabetic retinopathy (PDR) and retinopathy of prematurity (ROP)^3^. Although antiangiogenic therapies targeting the VEGF signaling cascade yield successes in many patients, the anti-VEGF biologics still have significant shortcomings due to non-responsive patients and drugs’ tachyphylaxis^4^. Therefore, there remains a critical need to uncover novel therapeutic targets to better understand and treat pathological ocular angiogenesis. Inflammatory signaling represents an appealing area in which to discover such therapeutic targets.

The nuclear factor (NF)-κB constitutes a family of inducible transcription factors that mediate the expression of a variety of genes involved in inflammation, cell growth and development, angiogenesis, and other pathways^5^. The five NF-κB family members, which include RelA/p65, RelB, c-Rel, p50/p105 (NF-κB1), and p52/p100 (NF-κB2), share the same REL homology domain (RHD) that is responsible for DNA binding and dimerization^6^. These transcription factors dimerize to regulate gene expression in two distinct NF-κB signaling pathways: the canonical pathway and the non-canonical pathway^7^. The canonical pathway, characterized by its quick and transient activity, is activated by cytokines, growth factors, and ligands of pattern recognition receptors (PRRs), TNF receptor (TNFR) superfamily members, T-cell receptor (TCR), and B-cell receptor^8^. Following the activation of the canonical pathway, a series of phosphorylation cascades involving IκB kinase (IKK) and the inhibitor of nuclear factor kappa B alpha (IκBα) leads to the translocation of p65/p50 into the nucleus, where they bind the promoters of their target genes and activate gene transcription^8^. On the other hand, the activation of the non-canonical pathway is mediated by the RelB/p52 complex and occurs in response to stimuli from ligands of receptors such as lymphotoxin β receptor (LTβR), BAFF receptor (BAFFR), CD40 and receptor activator of NF-κB (RANK). This pathway is involved in biological functions such as lymphoid organ development, B-cell survival and maturation, and bone metabolism^9^.

NF-κB, particularly the prototypical subunit p65, is extensively involved in the pathogenesis of different diseases. Given its critical role in inflammation, NF-κB contributes to the development and/or progression of conditions such as asthma, arthritis, inflammatory bowel disease, atherosclerosis, Alzheimer’s disease, cancer, and diabetes. In some cancers, the constitutive activation of NF-κB correlates with poor clinical course and outcomes^10^. Similarly, NF-κB activation controls the expression of proangiogenic genes, whose aberrant expression underlies the pathology of certain diseases^11–13^. Many studies have investigated the association of nvAMD with NF-κB in clinical samples and in AMD experimental models in vitro and in vivo. They show that activation of NF-κB induced inflammatory signaling and upregulated tumor necrosis factor α (TNF-α) and VEGF in RPE and other cells^14–25^. Thus, exploring key therapeutic druggable targets that directly mediate NF-κB regulation in AMD pathology, and their potent small molecule inhibitors would be highly valuable in the discovery of new therapeutics targeting nvAMD.

Previously, we identified protein arginine methyltransferase 5 (PRMT5) as a novel activator of NF-κB in cancer^26,27^. We speculated that PRMT5 could also play an important role in ocular neovascularization through its regulation of NF-κB. A type II arginine methyltransferase, PRMT5 belongs to the PRMT superfamily, and was initially named Janus-kinase binding protein (JBP1)^26^. PRMT5 is a multifunctional protein well-known for methylating histones to regulate their transcriptional activities. To date, PRMT5 has been shown to play a role in tumorigenesis and is overexpressed in multiple types of cancers including colon^27,28^, lung^29^, liver^30^, pancreas^27,31^, kidney^32^, brain^33^, and others. However, the role of PRMT5 in ocular neovascular diseases has never been examined before. PRMT5 is also known to have a critical role in the dimethylation of p65 (i.e., p65me2) and plays a role in hyperactivation of NF-κB, promoting NF-κB mediated cell signaling pathways^34^. Given this proinflammatory role, we expect the activity of PRMT5 on NF-κB activation to be increased in the setting of ocular neovascularization. Thus, targeting PRMT5 by novel inhibitors might render a beneficial therapeutic effect.

We developed a high throughput AlphaLISA screen approach and identified a potent small molecule inhibitor of PRMT5, PR5-LL-CM01. PR5-LL-CM01 reduced pancreatic and colorectal tumor phenotypes by inhibiting the activation of NF-κB and its downstream targets^27,35^. PR5-LL-CM01 demonstrated binding affinity to the active site of PRMT5 in silico, and it displayed anti-tumor effects in both in vitro and in vivo systems of pancreatic ductal adenocarcinoma (PDAC) and colorectal cancer (CRC) superior to the commercial inhibitor of PRMT5, EPZ015666^27^. We therefore propose PR5-LL-CM01 as a potential means to inhibit ocular neovascularization.

In this study, we show that PRMT5 is highly expressed in human and murine choroidal neovascularization and its genetic or chemical inhibition reduces proliferation, tube formation, NF-κB activation, and the expression of its target genes in ocular endothelial cells including human retinal microvascular endothelial cells (HRECs) and induced pluripotent stem-cell (iPSC) derived choroidal endothelial cells (iCEC2). Moreover, PR5-LL-CM01 blocked cell cycle progression at G_1_/S-phase in a dose-dependent manner in these cells. Collectively, our data suggest that PR5-LL-CM01 can be explored as a novel treatment lead for targeting PRMT5-mediated inflammation and angiogenesis in ocular neovascular diseases.

## Materials and methods

### Animals

All animal studies were approved by the Institutional Animal Care and Use Committee, Indiana University School of Medicine and the “Use of Animals in Ophthalmic and Visual Research” guidelines of the Association for Research in Vision and Ophthalmology and ARRIVE guidelines were followed. Wild-type C57BL/6J mice (female, 7 weeks of age) were purchased from Jackson Laboratory (Bar Harbor, ME, USA) and housed under standard conditions^36^ at the Laboratory Animal Resource Center, Indiana University School of Medicine.

### Laser-induced choroidal neovascularization (L-CNV) model

L-CNV was done as described before^37,38^ with minor modifications in laser power and duration. Briefly, mice were anesthetized by intraperitoneal injections of ketamine hydrochloride (80 mg/kg) and xylazine (10 mg/kg). The pupils of the eyes were dilated using tropicamide (1%) and phenylephrine (2.5%) and the eyes were exposed to laser treatment with 270 mW power pulses of the Micron IV laser injector (Phoenix Research Labs, Pleasanton, CA, USA) using 532 nm wavelength, 70 ms duration and 50 µm spot size. Delivered power was approximately 92 mW. Mice were euthanized and eyes removed at days 1, 7 and 14. The untouched control mice underwent enucleation at the end of the experiment on day 14. Eyes were fixed in 4% paraformaldehyde (PFA).

### Flat-mount staining

The RPE/choroids and retinas were dissected from the fixed eyes and prepared as flat mounts for PRMT5 and isolectin B4 (IB4) vasculature staining. In brief, the retina and choroid were fixed again with 4% PFA overnight at 4 °C. The tissues were then washed with phosphate-buffered saline (PBS, 1×) twice for 10 min each and permeabilized in blocking buffer containing 5% bovine serum albumin (BSA) and 0.5% Triton X-100 in PBS for 70 min. After blocking, the tissues were stained with isolectin B4 from *Griffonia simplicifolia* (GS-IB4) and anti-PRMT5 (see Supplementary Table S1 for details on the antibodies used) prepared in antibody blocking solution containing 0.5% BSA (Fisher, Pittsburgh PA, USA #BP9703-100) and 0.5% Triton X-100 (Sigma-Aldrich, St. Louis, MO, USA #T9284) for 48–72 h at 4 °C in a shaker. Four times for 15 min each, the tissues were washed with 1× PBS (Thermo Scientific, Waltham, MA, USA #10010-023) and incubated overnight in a shaker protected from light at 4 °C with respective detection reagents (DyLight 488-conjugated streptavidin, Invitrogen #21832; and Alexafluor 555-conjugated goat anti-rabbit antibody prepared 1:400 in antibody blocking solution. Then the tissues were washed with 1× PBS for four times, 15 min each and finally the immunostained tissues were flat mounted on glass slides and cover-slipped using Fluoromount-G (Southern Biotechnology, Birmingham, AL, USA). Until imaging, the flat mounts were protected from light and stored at 4 °C. The immunostained L-CNV lesions were imaged by confocal microscope (LSM700, Carl Zeiss, Thornwood, NY, USA) using 20× objective and Z-stack optical sectioning.

### Immunostaining

Deidentified human donor eyes from nvAMD patients and aged controls were sourced from the National Disease Research Interchange (NDRI, PA, USA), which obtains informed consent for donor materials following the principles stated in the WMA Declaration of Helsinki and the Department of Health and Human Services Belmont Report. The use of deidentified, decedent eyes was reviewed and designated “Not Human Subjects Research” by the Indiana University Institutional Review Board. The sections of the eye were deparaffinized, rehydrated and underwent antigen unmasking by heat-induced epitope retrieval in 1× citrate buffer (pH 6.0; Thermo Scientific, Waltham, MA, USA #AP-9003-500). The sections were rinsed with PBS and blocked for 2 h at room temperature using 10% normal donkey serum (NDS; Abcam, #ab7475) prepared in 1% BSA in PBS, then incubated with primary rabbit anti-PRMT5 and rabbit IgG control antibodies prepared in 10% NDS plus 1% BSA overnight at 4 °C (Supplementary Table S1). Sections were washed thrice with PBS-T (PBS + 0.025% Tween-20) for 5 min each. They were then incubated with Alexafluor 555-conjugated goat anti-rabbit secondary antibody for 1 h in a dark humidified chamber at room temperature, followed by washing thrice with PBS-T for 5 min each. Finally, the sections were dehydrated through an ethanol series and mounted with Vectashield mounting medium with the nuclear stain, DAPI. Using the 20× objective of a confocal microscope (LSM700, Carl Zeiss, Thornwood, NY, USA), the immunostained sections were imaged. The staining intensity of PRMT5 on these sections was quantified using ImageJ and the mean fluorescence intensity (MFI) of the region of interest (ROI; RPE or retina) was calculated by dividing the mean intensity by the number of pixels. To calculate the final MFI, the MFI of the ROI was subtracted from the MFI of a background area to assess if the nvAMD sections had PRMT5 staining intensity at levels contrasting to control sections analyzed.

The mouse eyes harvested after L-CNV induction at different days along with the eyes from untouched control mice were fixed in 4% PFA (Thermo Fisher Scientific, #43368) for 16 h at 4 °C. Dissected choroid and retina were embedded in optimal cutting temperature compound and cryosectioned to 5 µm thickness. The same immunostaining protocol described above was followed excluding deparaffinization, rehydration and antigen unmasking steps. The reagents (Supplementary Table S1) used for mouse cryosections were as follows: primary rabbit anti-PRMT5/rabbit IgG control, GS-IB4 and secondary Alexafluor 555-conjugated goat anti-rabbit antibody and DyLight 488-conjugated streptavidin (1:400 dilution). The mounted mouse sections were imaged on the LSM700 confocal microscope with a 20× objective.

### Immunoblotting for tissues

Immunoblotting from the mouse eye lysates was performed as described before^39^. Briefly, retina and choroid tissues dissected out from the L-CNV and untouched control mice (four eyes) were lysed for 20 min on ice in radioimmunoprecipitation assay (RIPA) buffer (Sigma #R0278) with protease inhibitors (cOmplete mini, #04693159001, Roche, Indianapolis, IN, USA) and phosphatase inhibitors (PhosSTOP, #04906837001, Roche). The samples were homogenized and centrifuged at 12,000×*g* for 15 min at 4 °C. Supernatants (lysates) were separated, and protein concentrations were determined using a bicinchoninic acid (BCA) protein assay. Equal amounts of protein (25–35 µg) from retina and choroid samples were resolved by 10% SDS-PAGE and transferred onto polyvinylidene fluoride (PVDF) membranes (Millipore, Burlington, MA, USA). Antibodies against PRMT5 and β-actin were used to detect the proteins (Supplementary Table S1) and secondary antibodies were anti-rabbit, IgG peroxidase conjugated and anti-mouse, IgG peroxidase conjugated. All of the antibody dilutions were prepared in Tris-buffered saline containing 2.5% BSA. Amersham ECL prime immunoblotting detection reagents (#RPN2236, GE Healthcare, Chicago, IL, USA) were used to detect immunoreactive bands on a ChemiDoc MP imaging system (Bio-Rad, Hercules, CA, USA).

### Cell culture

HRECs were purchased from Cell Systems (Kirkland, WA, USA) and grown in endothelial basal medium (EBM-2) in combination with an endothelial cell growth medium (EGM-2) bullet kit (Lonza, Walkersville, MD, USA). iCEC2 cells were a kind gift from Dr. Robert F. Mullins’ laboratory^40^ at the University of Iowa and grown in endothelial cell growth medium (R&D Systems, Minneapolis, MN, USA). iCEC2 cells contain a temperature-sensitive hypomorphic T antigen and actively proliferate at 33 °C, at which stocks were maintained, while growth slows at 37 °C, at which functional analyses were conducted. All cells were tested for mycoplasma contamination regularly.

### Construction of stable cells

FLAG-tagged WT-PRMT5 cDNA or FLAG-tagged WT-p65 cDNA was amplified by reverse transcription from total mRNA derived from 293 cells^34^. The sequences of WT-PRMT5 (NCBI sequence reference: NM_006109.3) and WT-p65 (NCBI sequence reference: NM_021975) were confirmed via DNA sequencing and then cloned into respective pLVX-IRES-puro vectors (https://www.takarabio.com/documents/Vector%20Documents/pLVX-IRES-Puro%20Vector%20Information.pdf). Lentiviral vectors expressing five different PRMT5-directed shRNAs (target set RHS4533-EG10419), and the universal negative control, pLKO.1 (RHS4080) were purchased from Open Biosystems (Dharmacon, Lafayette, CO, USA). To generate stable cells, the lentiviral plasmid containing the DNA of interest or shRNAs targeting *PRMT5* exons were transfected into a 293T packaging cell line to produce viruses. HRECs or iCEC2 cells were then infected with these viruses and further selected with 1 µg/ml of puromycin, as the lentiviral vector construct includes a puromycin resistance gene. Expression of the respective constructs was confirmed using immunoblotting with specific antibodies. Cells were used for further experiments, with HRECs used between passages 5 and 7.

### Proliferation assays

HRECs and iCEC2 cells overexpressing PRMT5 and shPRMT5 knockdown cell lines were plated at 2 × 10^4^ cells/well in a 6-well plate. Cells were seeded in triplicate and counted on different days using a hemocytometer. For compound effects on proliferation, HRECs and iCEC2 cells were evaluated as described earlier^37,38,41^. In brief, the cells were seeded in 96-well black plates with clear bottom at a density of 2.5 × 10^3^ with 100 µl of growth media and incubated for a day. After 24 h, 1 µl of PR5-LL-CM01 (0.1 nM to 100 µM) or vehicle DMSO (at 1% final concentration to the cells) was added, and the plates were incubated for 44 h. To each well of the plates, 11.1 µl of Alamar blue reagent was added and the readings were taken after 4 h in a Synergy H1 plate reader (BioTek, Winooski, VT, USA) with 560 nm (excitation) and 590 nm (emission) wavelengths. Using GraphPad Prism 9.0, GI_50_ (growth inhibitory concentration) was calculated for PR5-LL-CM01. To assess VEGF-induced cell growth of HRECs, the cells (2.5 × 10^3^) were grown in EBM-2 + 5 ng/ml recombinant human VEGF-A (583702, Biolegend, San Diego, CA, USA), treated with PR5-LL-CM01 (0.1 nM to 100 µM) or vehicle DMSO and assayed as detailed above.

### Flow cytometry cell cycle analysis

The analysis of the cell cycle in HRECs and iCEC2 cells was assessed as described before^38^. Briefly, cells were grown in 6 well plates (2 × 10^6^) and at 70% confluency, the cells were treated with PR5-LL-CM01 (0.1 µM, 1 µM and 10 µM) or vehicle DMSO (1% final concentration to the cells) for 24 h. The cells were washed with PBS twice and fixed in 70% ethanol. Prior to analysis, the cells were washed with PBS for two times, the cell pellets resuspended in propidium iodide (PI) staining solution (20 µg/ml) prepared in 1× PBS containing 100 µg/ml of RNAse A and 0.1% Triton X-100 and incubated at room temperature for 30 min. The cells were then analyzed on an Attune NxT flow cytometer (Thermo Fisher Scientific, Waltham, MA, USA). The single cell population was analyzed by area histograms and cell cycle profiles were created using Modfit software (v. 5.0, Verity Software House, Topsham, ME, USA). Pulse shape analysis was performed to eliminate any debris, doublets and aggregates from the whole cell population analyzed.

### Matrigel tube formation assay

The tube formation ability of HRECs, iCEC2 cells and the respective stable cells was assessed as previously described^37,38^. Briefly, the wells of a 96 well plate were precoated with Matrigel (50 µl), which was allowed to solidify at 37 °C for 20 min. The cells were seeded at a density of 1.5 × 10^4^ in growth medium (100 µl) containing 1 µl/well PR5-LL-CM01 (0.1 µM, 1 µM and 10 µM) or vehicle DMSO (1% final concentration in the wells). The stable cells were seeded at the same density over Matrigel basement membrane. Tube formation was monitored every 2 h, and after incubating the cells for 8 h at 37 °C and 5% CO_2_, each well was photographed using a brightfield microscope (4× objective), and the measurements of formed tubes, meshes, branches, and segments were analyzed using AngiogenesisAnalyzer plugin in ImageJ software (v. 1.8.0; http://image.bio.methods.free.fr/ImageJ/?Angiogenesis-Analyzer-for-ImageJ.html).

### Immunoblotting for cells

HRECs and iCEC2 cells were pelleted post treatment with PR5-LL-CM01 (0, 1.5, 3, or 6 µM respectively for 24 h) in phosphate buffered saline (PBS). Pellets were lysed with lysis buffer [10 mM Tris–Cl pH 8.0, 1 mM EDTA, 1% Triton X-100, 0.1% sodium deoxycholate, 0.1% SDS (sodium dodecyl sulfate), 14 mM NaCl, 1 mM phenylmethylsulfonyl fluoride]. Protein concentration for each sample was determined using Protein Assay Reagent (Bio-Rad, Hercules, CA, USA). Equal protein concentrations were run on 10% SDS-PAGE and transferred to a PVDF membrane (Thermo Fisher Scientific). Membranes were exposed to anti-p65, anti-p65me2, anti-FLAG, anti-PRMT5, and anti-actin and their respective secondary antibodies, mouse or rabbit respectively, based on manufacturer’s instructions (Supplementary Table S1). Protein signal was detected using enhanced chemiluminescence (ECL) reagent (PerkinElmer) and quantified where indicated using ImageJ.

### NF-κB luciferase assay

The NF-κB luciferase lentiviral construct pLA-NFκBmCMV-luc-H4-puro^42^, an NF-κB reporter lentiviral vector, consists of a firefly luciferase reporter gene under the control of a minimal (m)CMV promoter and six NF-κB-responsive elements from the immunoglobulin light chain gene (kind gift from Peter Chumakov, Russian Academy of Sciences, Moscow, Russia). This plasmid was introduced into the respective cells using Lipofectamine™ LTX Reagent and PLUS Reagents (Thermo Fisher Scientific). Luciferase activity was measured after 48 h (with or without drug treatment) using Reporter Lysis Buffer kit (Promega, Madison, WI) per manufacturer’s instructions and a Synergy H4 plate reader.

### Quantitative reverse-transcription polymerase chain reaction (qRT-PCR)

Following treatment with PR5-LL-CM01 for 24 h, total RNA was isolated from the respective cells using TRIzol. First strand complementary DNA was generated using SuperScript III First-Strand Synthesis Kit (Invitrogen, Carlsbad, CA). *GAPDH* was selected as the housekeeping gene for normalization; each gene was run along with *GAPDH*, and the difference between threshold cycles (C_T_) was designated as ΔC_T_. ΔΔC_T_ is the difference between their respective controls. qPCR was executed using FastStart Universal SYBR Green kit (Roche, Indianapolis, IN). Primers were designed using the Primer Express 3.0 software (Thermo Fisher Scientific). Primers used are listed in Supplementary Table S2.

### Statistical analyses

The data were analyzed using GraphPad Prism software (version 9.2.0). Unpaired Student’s *t*-test with Welch’s correction was used for comparing two means, while one-way ANOVA with Dunnett’s post hoc tests was used for comparing more than two means. Two-sided p values < 0.05 were considered statistically significant.

## Results

### PRMT5 is highly expressed in nvAMD

**Figure 1:**
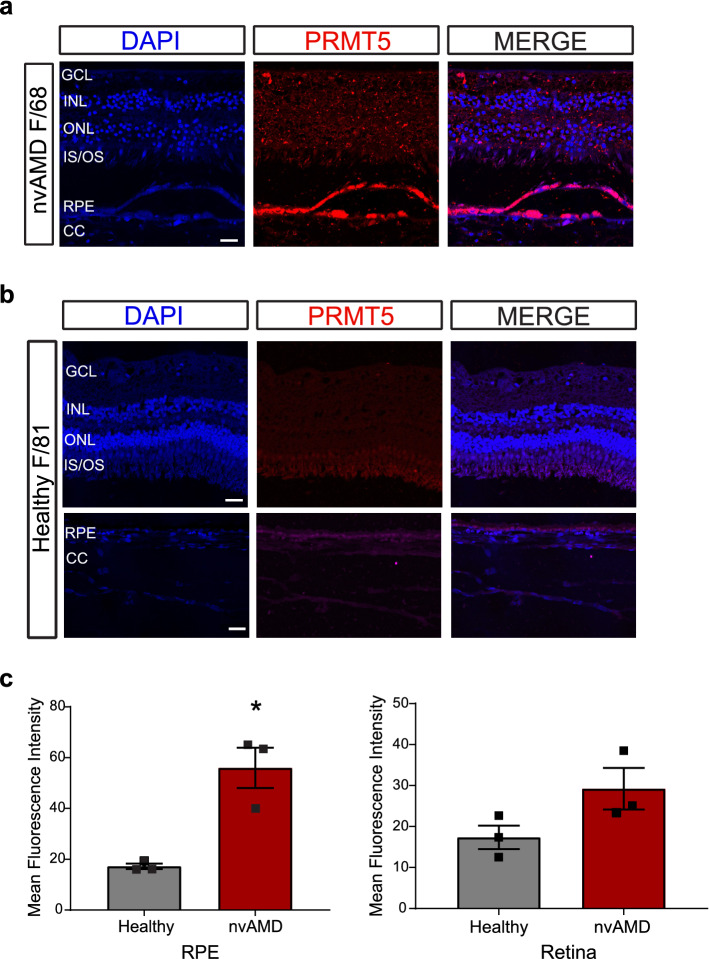
PRMT5 is highly expressed in neovascular age-related macular degeneration (nvAMD). PRMT5 immunostaining on sections of eyes from human (**a**) nvAMD patient (68 year old female) and (**b**) control (81 year old female), where DAPI (blue) shows the nuclei of the cells and red indicates PRMT5 expression in different layers of the retina, and in the RPE/choroid complex. Higher expression of PRMT5 is observed in the retinal pigment epithelium (RPE)/choroid in the eye with nvAMD. Representative images shown from n = 3 nvAMD patients and controls (see Supplementary Fig. S1). (**c**) Quantification of PRMT5 mean fluorescence intensity (MFI) in RPE and retina of nvAMD vs controls. Mean ± SEM, n = 3. *p < 0.05, Student’s *t*-test with Welch’s correction. Scale bars = 20 µm. GCL, ganglion cell layer; INL, inner nuclear layer; ONL, outer nuclear layer; IS/OS photoreceptor inner/outer segments; CC, choriocapillaris.

To investigate the potential role of PRMT5 in ocular neovascularization, we first sought to examine its expression in postmortem eyes from human nvAMD patients. Sections of human nvAMD eyes in comparison with healthy control eyes showed PRMT5 expression in all the layers of the retina, with especially high expression seen in the RPE-choroid where neovascularization originates in nvAMD (Fig. 1a,b). Quantification of staining intensity corroborated this observation: the intensity of PRMT5 in RPE and retina was markedly higher in nvAMD samples than the healthy control samples (Fig. 1c). Also, AMD eyes compared to healthy control eyes clearly display the distinctive degenerative phenotypes, including the loss of inner or outer segments of photoreceptors and disruption in the retinal nuclear layer architecture (Fig. 1a), although these changes and the pattern of PRMT5 expression vary among the AMD eyes we studied (Supplementary Fig. S1).

### PRMT5 is highly expressed in murine L-CNV

**Figure 2:**
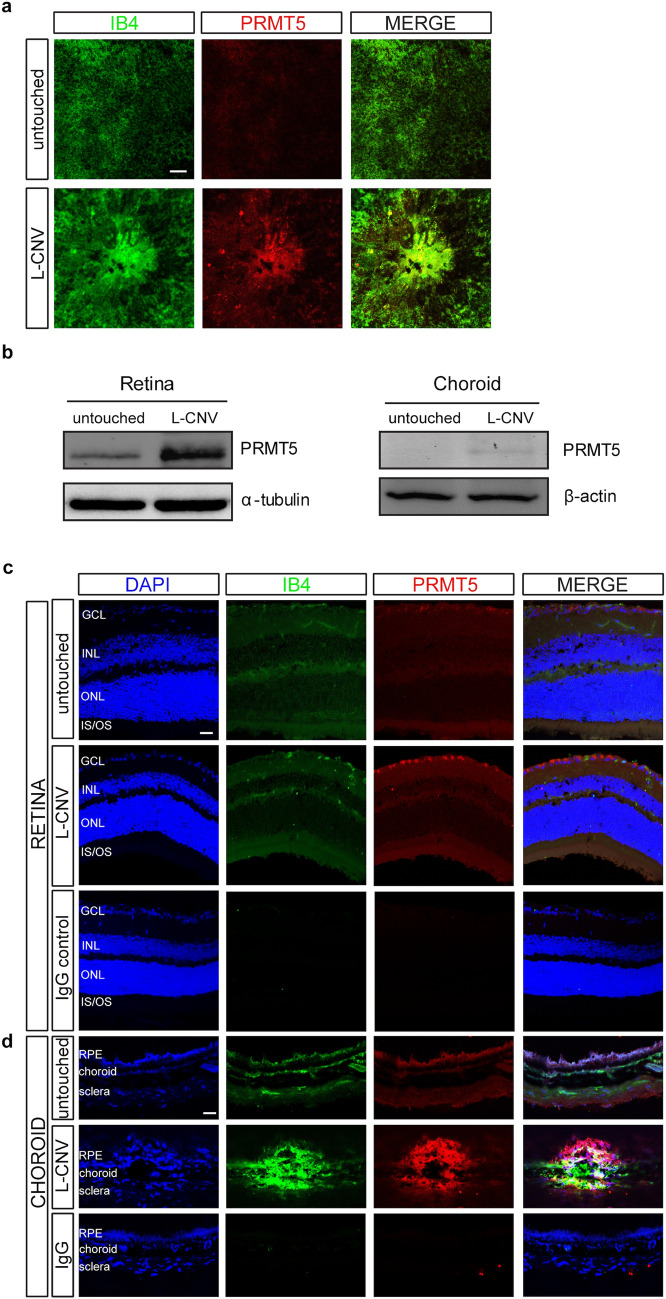
PRMT5 expression in murine laser-induced choroidal neovascularization (L-CNV). (**a**) Flat mount staining of L-CNV choroids, showing the expression of PRMT5 (red) in and around the neovascular lesion in the choroid that underwent laser treatment compared to untouched control. (**b**) Immunoblot, showing PRMT5 is highly expressed in the retina and choroid of the L-CNV mouse eyes compared to the untouched control eyes. See also Supplementary Fig. S2. (**c**,**d**) Cryosections of the L-CNV (**c**) retina and (**d**) choroid, showing PRMT5 expression in different layers of the retina, including the ganglion cell layer (GCL), inner plexiform layer (IPL), outer plexiform layer (OPL), and in the inner and outer segments (IS/OS) of photoreceptors, and in the retinal pigment epithelium (RPE)-choroid complex where neovascularization is observed (isolectin B4 [IB4] staining, green). Higher expression is observed in the GCL and in the RPE-choroid in L-CNV compared to untouched.

Given the expression of PRMT5 in nvAMD, we next asked whether PRMT5 was similarly upregulated in experimental CNV. We studied this using a mouse model of L-CNV, wherein the mouse eyes were harvested at 7 days post laser treatment and assessed for the expression of PRMT5 in the retina and choroid. Overexpression of PRMT5 was observed in and around the L-CNV lesions in choroidal flat mounts compared to the flat mounts prepared from untouched control eyes (Fig. 2a). Immunoblot in comparison with untouched control eyes showed relatively increased levels of PRMT5 both in retina and choroid (Fig. 2b, Supplementary Fig. S2) in the L-CNV samples. Immunostaining of PRMT5 on mouse eye cryosections revealed high expression and localization of PRMT5 in the ganglion cell layer (GCL) of the retina and in the RPE-Bruch’s membrane (BM)-choroid complex in L-CNV, although some expression was seen throughout the retina (Fig. 2c,d).

### PRMT5 regulates endothelial cell proliferation

**Figure 3:**
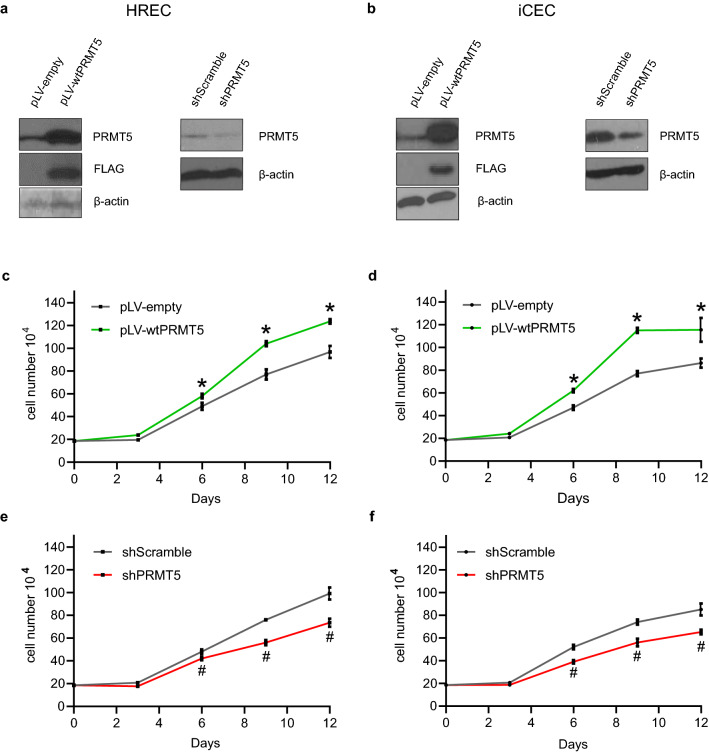
PRMT5 overexpression promotes cell growth. (**a**) Immunoblot, showing FLAG-tagged wild-type (wt) PRMT5 successfully overexpressed (*left panel*) or knocked down (*right panel*) in HRECs. (**b**) Immunoblot, showing FLAG-tagged wtPRMT5 successfully overexpressed (*left panel*) or knocked down (*right panel*) in iCEC2 cells. See also Supplementary Fig. S3. (**c**–**f**) Effect of PRMT5 on cell proliferation. Overexpression of wtPRMT5 promoted cell growth in HRECs (**c**) and iCEC2 cells (**d**), while shPRMT5 knockdown had the opposite effect in HRECs (**e**) and iCEC2 cells (**f**). Mean ± SEM, n = 3–4 biological replicates. *p < 0.05 wtPRMT5 vs. pLV-empty; ^#^p < 0.05, shPRMT5 vs. shScramble, unpaired Student’s *t*-tests with Welch’s correction.

Due to the expression profile of PRMT5 in CNV, we generated PRMT5 overexpression and shPRMT5 knockdown in HRECs and iCEC2 cells. We were able to successfully overexpress a FLAG-tagged PRMT5 protein in HRECs and iCEC2 cells compared to a vector control and to knock down PRMT5 compared to shScramble controls (Fig. 3a,b; Supplementary Fig. S3). These cells were then used in future experiments. As NF-κB is an important regulator for cell growth, we first performed cellular proliferation assays (Fig. 3c–f). Overall, overexpression of PRMT5 increased cellular growth compared to the vector control, while shRNA knockdown of PRMT5 decreased growth compared to the shScramble control. These data suggest PRMT5 plays an important role in promoting ocular endothelial cell proliferation.

### PRMT5 inhibitor PR5-LL-CM01 exhibits antiangiogenic properties in ocular endothelial cells

**Figure 4:**
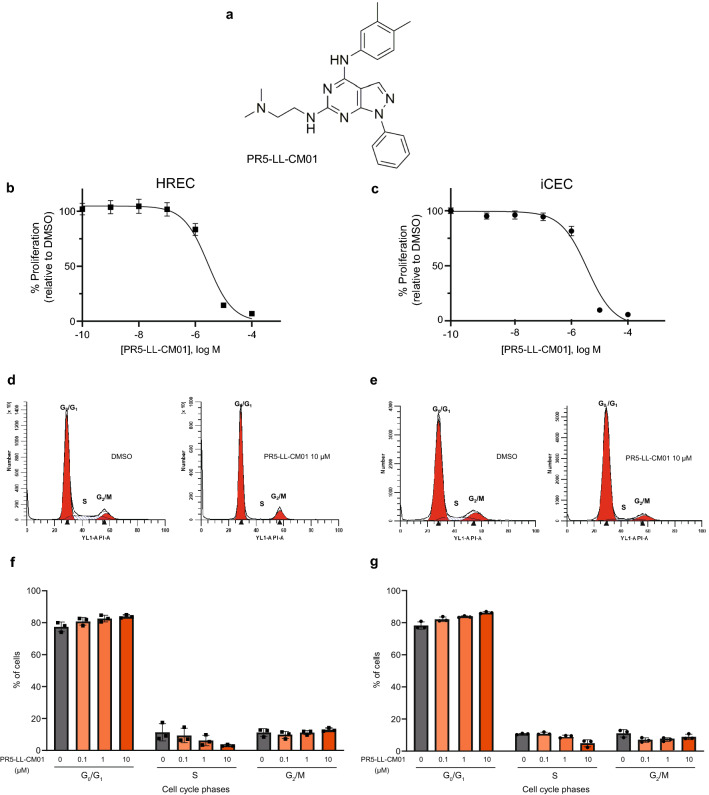
PR5-LL-CM01 is antiangiogenic in vitro. (**a**) Structure of PRMT5 inhibitor PR5-LL-CM01^27^. (**b**,**c**) PR5-LL-CM01 is antiproliferative at 48 h of treatment on HRECs (**b**) and on iCEC2 cells (**c**). Mean ± SEM, n = 3 technical replicates. Representative data from three biological replicates. PR5-LL-CM01 dose-dependently decreases cells in S-phase and increases cells in G_0_/G_1_ after 24 h of treatment in (**d**,**f**) HRECs, and in (**e**,**g**) iCEC2 cells. Mean ± SEM of percentage of cells, n = 3 biological replicates.

PR5-LL-CM01 (Fig. 4a) has been identified in our lab as an effective PRMT5 inhibitor which downregulates NF-κB activity^27^. Previously, the effect of PR5-LL-CM01 has been shown only in the context of cancer^27^. Here, we further studied PR5-LL-CM01 in HRECs and iCEC2 cells. Relative to DMSO, PR5-LL-CM01 dose-dependently reduced the proliferation of these cells in complete medium in a low micromolar range, GI_50_ = 2.42 µM in HRECs and 2.98 µM in iCEC2 cells (Fig. 4b,c). Moreover, PR5-LL-CM01 also dose-dependently reduced HREC proliferation in response to VEGF alone with GI_50_ = 1.14 µM. The antiproliferative effect was evident by the cell cycle arrest in these endothelial cells, where increasing concentrations of PR5-LL-CM01 decreased cells in S phase in comparison to the cells treated with 1% DMSO (Fig. 4d–g). Further, a concomitant increase in the cell populations was noted in the G_0_/G_1_ phase of the cell cycle on increasing doses of PR5-LL-CM01, suggesting a concentration-dependent inhibition of cell transition from G_0_/G_1_ to S-phase.

### PR5-LL-CM01 decreases PRMT5-mediated NF-κB activation and its downstream target gene expression

**Figure 5:**
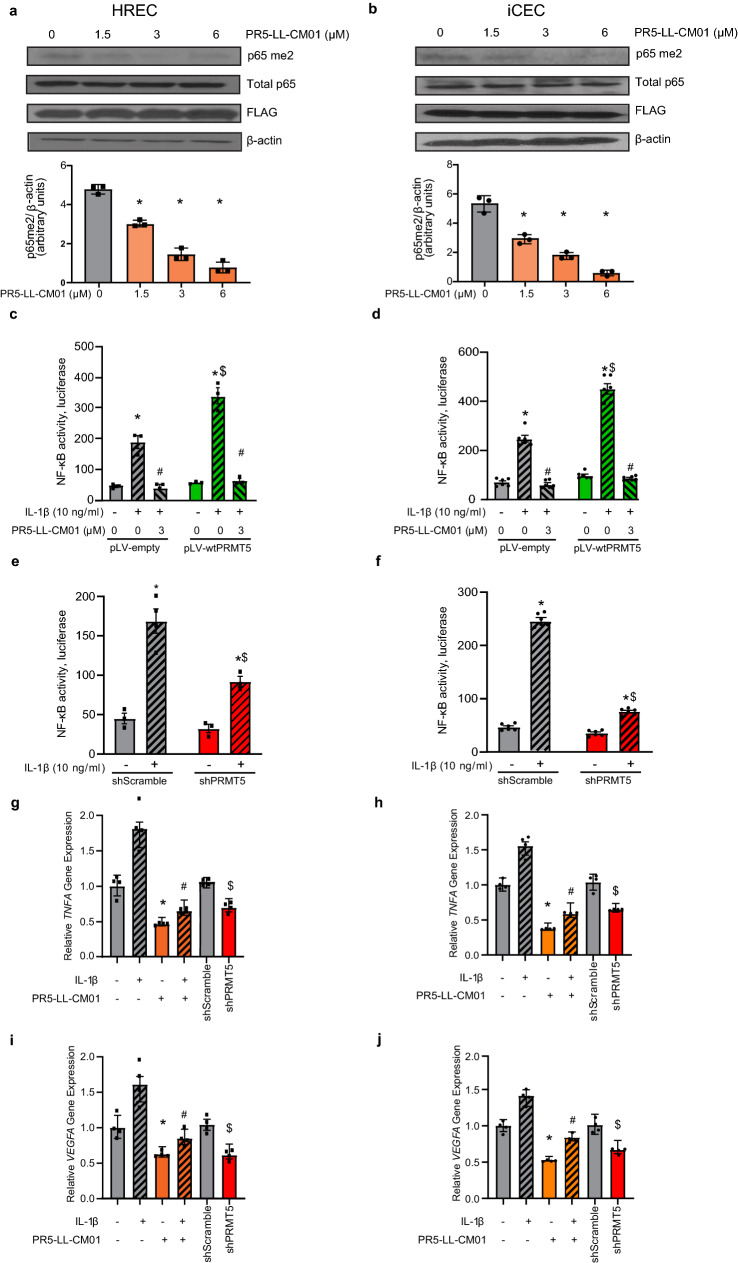
Treatment with PR5-LL-CM01 decreases p65me2, NF-κB activity, and NF-κB target gene expression in HRECs and iCEC2 cells. (**a**,**b**) Immunoblots and quantification, indicating inhibition of p65-R30me2 in a stepwise manner with increasing concentrations of PR5-LL-CM01 in HRECs (**a**) or iCEC2 cells (**b**) overexpressing FLAG-tagged wt-p65. Lower panels show ImageJ quantification of p65-R30me2 relative to loading control (β-actin) for three independent immunoblots. *p < 0.05 vs. 0 µM PR5-LL-CM01 group. See also Supplementary Fig. S4. (**c**,**d**) NF-κB luciferase assay. wtPRMT5 overexpressing HRECs (**c**) or iCEC2 cells (**d**) were stimulated with 10 ng/ml IL-1β ± 3 µM PR5-LL-CM01 for 4 h. PRMT5 overexpression augmented NF-κB induction upon IL-1β treatment, while PR5-LL-CM01 inhibitor treatment reduced this effect. *p < 0.05 vs. IL-1β untreated group; ^#^p < 0.05 vs. IL-1β-induced group; ^$^p < 0.05 vs. pLV-empty + IL-1β-treated group. (**e**,**f**) Both shScramble and shPRMT5 HRECs (**e**) or iCEC2 cells (**f**) were stimulated with 10 ng/ml IL-1β for 4 h. shPRMT5 inhibited NF-κB activity upon IL-1β treatment. *p < 0.05 vs. IL-1β untreated group; ^$^p < 0.05 vs. shScramble + IL-1β-treated group. n = 3–4 technical replicates. (**g**,**i**) HREC vector cells or (**h**,**j**) iCEC2 vector cells were stimulated with 10 ng/ml IL-1β ± 3 µM PR5-LL-CM01 for 4 h. PR5-LL-CM01 significantly decreased the expression of *TNFA* (**g**,**h**) and *VEGFA* (**i**,**j**) mRNA. Likewise, shPRMT5 reduced *TNFA* and *VEGFA* levels. *p < 0.05 vs. IL-1β untreated and PR5-LL-CM01 untreated group; ^#^p < 0.05 vs. IL-1β-induced group, and PR5-LL-CM01 untreated group; ^$^p < 0.05 vs. shScramble group. Mean ± SD, n = 3–4 biological replicates. Unpaired Student’s t-test with Welch’s correction was used for comparing two means, and one-way ANOVA with Dunnett’s post hoc tests was used when comparing more than two means.

As PR5-LL-CM01 is known to inhibit NF-κB activity by downregulating PRMT5 activity in cancer^27^, we sought to determine if PR5-LL-CM01 has a direct effect on the dimethylation of the p65 subunit of NF-κB (p65me2), using p65 overexpressing HRECs and iCEC2 cells. Upon treatment with PR5-LL-CM01, p65me2 was decreased in a dose-dependent manner (Fig. 5a,b; Supplementary Fig. S4). This suggests PR5-LL-CM01 inhibited PRMT5-mediated p65me2.

Given the documented regulation of NF-κB by PRMT5^27^, we wished to determine if PRMT5 overexpression or shPRMT5 knockdown altered NF-κB activity via a luciferase assay and if this activity could be reduced with the addition of PR5-LL-CM01 in retinal and choroidal endothelial cells. Overexpression of PRMT5 significantly increased IL-1β-induced NF-κB activity, while addition of PR5-LL-CM01 dramatically reduced NF-κB activity in both HRECs (Fig. 5c) and iCEC2 cells (Fig. 5d). Furthermore, shPRMT5 knockdown decreased IL-1β-induced NF-κB activity compared to respective controls in both HRECs (Fig. 5e) and iCEC2 cells (Fig. 5f). To further evaluate the effect of PRMT5 inhibition on NF-κB target gene expression, we conducted qPCR analysis of well-known NF-κB target genes involved in inflammation and angiogenesis: *TNFA, VEGFA,* and *VEGFR2.* The expression of *TNFA* (Fig. 5g,h), *VEGFA* (Fig. 5i,j), and *VEGFR2* (Supplementary Fig. S5) were significantly decreased by either PR5-LL-CM01 treatment or shPRMT5 knockdown as compared to their respective control cells, in both HRECs (Fig. 5g,i; Supplementary Fig. S5a,c) and iCEC2 cells (Fig. 5h,j; Supplementary Fig. S5b,d).

Taken together, the above data suggest that PR5-LL-CM01 decreases PRMT5-mediated NF-κB activation and its downstream target gene expression, thus presenting promising therapeutic antiangiogenic and anti-inflammatory potential through NF-κB inhibition.

### PRMT5 knockdown reduces angiogenic tube formation in ocular endothelial cells

**Figure 6:**
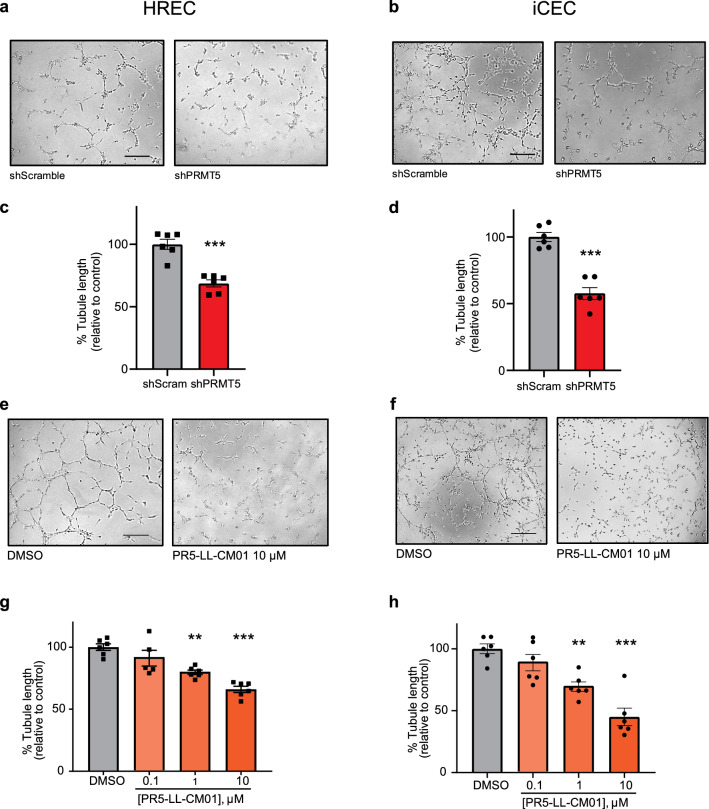
PRMT5 inhibition by shRNA knockdown or PR5-LL-CM01 treatment reduces tube formation in HRECs and iCEC2 cells. (**a**–**d**) Tube formation assay in HRECs (**a**,**c**) or iCEC2 cells (**b**,**d**) transduced with shScramble vector or shPRMT5 reveals that knockdown of PRMT5 reduces tube formation ability. ***p < 0.0001 vs. shScramble control, unpaired Student’s t-test with Welch’s correction. (**e**–**h**) Quantitative analysis of tube formation in HRECs (**e**,**g**) and in iCEC2 cells (**f**,**h**) demonstrates that PR5-LL-CM01 blocks tube formation in a dose-dependent manner. Mean ± SEM, n = 6–12 images. **p < 0.01; ***p < 0.001 vs. DMSO control, one-way ANOVA with Dunnett’s post hoc test. Representative data from three biological replicates. Scale bars = 500 µm. See also Supplementary Fig. S6.

Tube formation is an in vitro property of endothelial cells reflective of angiogenic potential in vivo. The Matrigel tube formation assay is a widely used, reproducible model system to study either the activation or inhibition of angiogenic pathways in vitro. Knockdown of PRMT5 in various cancer cells can suppress the protumorigenic functions of PRMT5^43–46^. Therefore, we sought to assess whether genetic or chemical inhibition of PRMT5 affects tube formation in HRECs and iCEC2 cells. PRMT5 knockdown (Fig. 6a–d, Supplementary Fig. S6a,b) in both cell types significantly reduced tube formation ability (measured by tube length, mesh, branch, and segment) compared with the shScramble control. Consistent with this, PRMT5 inhibition by PR5-LL-CM01 dose-dependently decreased tube formation in both cell types as well (Fig. 6e–h, Supplementary Fig. S6c,d). These data suggest that inhibition of PRMT5 by PR5-LL-CM01 is comparable with genetic knockdown and both can halt angiogenic properties of cells relevant to ocular neovascular diseases.

### Hypothetical model

**Figure 7:**
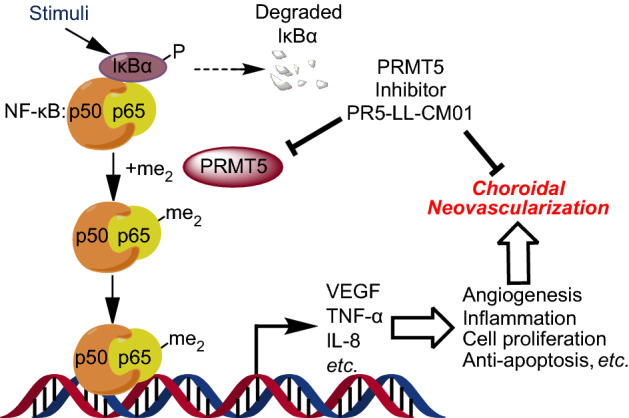
Hypothetical model. PRMT5 methylates and activates NF-κB. This results in the induction of NF-κB downstream genes, including cytokines, angiogenesis factors, chemokines, and antiapoptotic genes, whose functions are critical for inflammation and angiogenesis. Thus, using PR5-LL-CM01 to block the activity of PRMT5 will inhibit neovascularization-associated eye diseases.

Based on the findings from this study, we provide evidence that PRMT5 methylates and activates NF-κB in endothelial cells. This results in the induction of NF-κB downstream genes, known to include cytokines, angiogenesis factors, chemokines, and antiapoptotic genes, whose functions are critical for inflammation and angiogenesis. Thus, using PR5-LL-CM01 to block the activity of PRMT5 has potential to inhibit neovascularization-associated eye diseases (Fig. 7).

## Discussion

Understanding the molecular mechanisms of cellular angiogenic components is crucial for identifying new therapeutics targeting ocular neovascular diseases such as nvAMD, PDR, and ROP. We showed here for the first time that PRMT5 is a novel potential therapeutic target for nvAMD. In this study, we provide experimental evidence that high expression of PRMT5, as observed in the murine L-CNV model and human nvAMD tissues, can impact the regulation of proangiogenic and proinflammatory NF-κB signaling; inhibiting PRMT5 with a potent small molecule inhibitor, PR5-LL-CM01, offers potential therapeutic efficacy.

Our work adds to a body of evidence linking NF-κB signaling with neovascularization. Although the pathophysiology of nvAMD is multifactorial, NF-κB-mediated inflammation and activation of angiogenic VEGF signaling are vital processes that control neovascularization in the ocular system. Growing evidence has shown the influence of NF-κB signaling as a target for nvAMD^17,18,20,22^. As a master regulator, NF-κB regulates inflammation in the retina under conditions of stress through the transcriptional regulation of various cytokines and angiogenic factors. Such activation of NF-κB inflammatory signaling eventually results in VEGF upregulation^47^. Since the endothelial cell microenvironment is the target site of angiogenesis, previous studies have documented the role of increased TNF-α, activation of NF-κB, and higher VEGF levels in angiogenic endothelial cells including human umbilical vein endothelial cells (HUVECs)^48^ and human cardiac microvascular endothelial cells (HCMECs)^49^. In the context of ocular angiogenesis, the activation of NF-κB and the associated inflammatory responses stimulated by thrombin have been studied in HRECs in the milieu of diabetic retinopathy^50^. Studies on human RPE cells have shown increased VEGF levels stimulated either through TNF-α or NF-κB activation^19,51–53^. Wang et al.^25^ documented that neo-vessel genesis of choroidal endothelial cells may be caused by TNF-α facilitated NF-κB activation and VEGF production. However, there have not been studies on ocular endothelial cells targeting NF-κB activation triggered by PRMT5-mediated dimethylation.

We began our investigations by assessing the expression of PRMT5 in human AMD and the L-CNV model. This latter model is an extensively used, robust model system wherein the injury induced in Bruch’s membrane and RPE by laser treatment results in neovascularization akin to that observed in the human pathology in terms of position and appearance of choroidal neo-vessels^54,55^. L-CNV mouse eyes have high expression of TNF-α, and intravitreal injections of anti-TNF-α antibody treatment reduced the lesion volume induced in conjunction with lowered expression of VEGF in the RPE/choroid^19^. The role of NF-κB and its activation by TNF-α have been extensively studied in the murine L-CNV model^16,18,56^. Furthermore, recent work showed that NF-κB signaling activated microglial cells to drive angiogenesis in mice with L-CNV, and a competitive inhibitor of NF-κB kinase subunit β (IKKβ) diminished the lesion volume through inhibiting NF-κB signaling and VEGF-A expression^22^. In L-CNV, we observed higher expression of PRMT5 in retina and choroid, suggesting a potential correlation between PRMT5 and CNV. Increased PRMT5 expression in L-CNV may enhance NF-κB activation and proinflammatory cytokine production, as we have shown in progression of cancer and metastasis^27,34,57^. Further molecular evaluation of this hypothesis will be an interesting next step.

PRMT5 was also present throughout the retina and choroid in human eyes and increased in disease, possibly including endothelial and/or microglial cells activated during neovascularization in nvAMD. NF-κB mediates the regulation and function of proinflammatory genes in innate and adaptive immune cells^8^. Thus, it is possible that the presence of PRMT5 could be correlated with the characteristic increase of activated microglia and chorioretinal endothelial cells, as has been implicated before in early and late AMD^58,59^. It is worth noting that in murine L-CNV, the expression of PRMT5 was partially co-localized with retinal and choroidal vasculature, suggesting the incidence of proangiogenic events likely instigated by increased VEGF secretion and endothelial cell activation. However, we acknowledge that endothelial cells are not the sole players of proinflammatory cytokine and proangiogenic VEGF production. Innate immune cells of the retina such as microglia, infiltrating macrophages, and astrocytes can promote VEGF secretion and dysregulation in neovascularization. In the case of inflammation/injury in the retina, microglia and macrophages are recruited, amassed, and triggered in a VEGF-A dependent process to release proinflammatory cytokines^60^. Moreover, astrocytes secrete VEGF-A under hypoxia and promote vessel sprouting^61,62^. During acute retinal injury, astrocytes interact with reactive microglia and are responsible for neuroinflammation^63^, and thereby neurodegeneration of retinal cells. Future studies will explore PRMT5’s role in VEGF signaling in these other cell types, given the ubiquitous expression of PRMT5^64^.

Retinal ganglion cells (RGCs) and RPE cells safeguard the neurosensory retina^65,66^. For instance, stressed RGCs collapse the endogenous mechanism of neuroprotection and thus gradually affect the other cells of the retinal architecture^67^. RPE cells preserve the health of the choriocapillaris besides governing the outer blood-retinal barrier^68,69^. The RPE is also a primary producer of inflammatory signals, and the occurrence of chronic inflammatory processes leads to dysfunction of RPE^70^. Thus, the high expression of PRMT5 in the GCL of L-CNV mouse retina and in the RPE of mouse eyes and human AMD supports the notion that PRMT5 could have potential functional significance in the nvAMD-related neurodegeneration and RPE atrophy, though this speculation requires future experimental clarification. It also remains to be seen if PRMT5 inhibition may have deleterious effects on these cell types with high PRMT5 expression.

PRMT5 can regulate proliferation, differentiation, invasion, and migration of tumor cells^71^. Additionally, we have reported that PR5-LL-CM01, a promising inhibitor of PRMT5, has anti-tumor activity in vitro and in vivo in PDAC and CRC^27^. In the present study, we showed that inhibition of PRMT5 via PR5-LL-CM01 is anti-angiogenic, dose-dependently impeding key angiogenesis properties of proliferation and tubule formation^72,73^ in ocular (retinal and choroidal) endothelial cells. Likewise, PRMT5 blockade by shRNA knockdown reduced proliferation and tube formation in these cells, while overexpression of PRMT5 enhanced proliferation.

A number of earlier studies demonstrated that the activation of NF-κB has a direct link to cell cycle progression^74^. For instance, NF-κB inhibition impairs the progression of the cell cycle in HeLa cells^75,76^ and human glioma cells^77^. Therefore, we speculate that the observed G_1_/S-phase blockage induced by PR5-LL-CM01 in HRECs and iCEC2 cells might be, at least in part, associated with the PR5-LL-CM01 induced inhibition of PRMT5-mediated NF-κB activation. In the future, it would be interesting to further examine the detailed mechanism of cell cycle arrest mediated by PR5-LL-CM01.

To our knowledge, the mechanistic role of PRMT5 in endothelial cells, notably in ocular endothelial cell biology, has never been studied, although the dynamic role of PRMT5 in tumorigenesis, NF-κB methylation, and activation has been studied by us^27,34,35^ and others^78–80^. Therefore, we studied PRMT5-regulated NF-κB activity and the expression of its downstream target genes responsible for inflammation and angiogenesis, such as TNF-α, VEGF-A, and its cognate receptor VEGFR2 in retinal and choroidal endothelial cells. Using qPCR analysis, we confirmed that *VEGFA, VEGFR2* and *TNFA* genes were downregulated by PR5-LL-CM01 treatment in HRECs and iCEC2 cells. Particularly, the downregulation of both *VEGFA* and *VEGFR2* by PRMT5 inhibition suggests a novel mechanism for PRMT5 in promoting angiogenesis. Collectively, we demonstrate that inhibition of PRMT5 reduces NF-κB activity and downregulates target genes important for angiogenesis. The full spectrum of NF-κB target genes regulated by PRMT5 in endothelial cells remains to be explored. We acknowledge that PRMT5 also has other substrates (as seen in other cell types) given its important role as an arginine methyltransferase. PRMT5 substrates are involved in transcription, RNA processing, proliferation and metabolism^81^. Some of these substrates play important roles in cell activity such as EGFR, histones, and the critical tumor suppressor p53^81–83^. Histone methylation by PRMT5 enhances and suppresses genes via regulation of methylated transcription factors^81^. Furthermore, PRMT5 can regulate methylation of DNA repair protein Rad9 which is necessary for the control of cell cycle checkpoints^84^. It would be interesting to further investigate whether other potential PRMT5 substrates are also involved in nvAMD in the future.

Unsurprisingly, much effort has been made to develop NF-κB inhibitors. However, targeting specific pathways that regulate NF-κB has been the preferred approach rather than targeting NF-κB itself, because basal NF-κB activity is vital to normal cellular functions and immune responses. To date, there are very few FDA-approved NF-κB inhibitors, and most focus on blood cancers; *e.g.* bortezomib is a proteasome inhibitor not specific to NF-κB, and used on mantle cell lymphoma, multiple myeloma, and Waldenström macroglobulinemia. Thus, there remains an urgent need to develop NF-κB inhibitors that target clinical indications other than blood cancer, such as ocular neovascular diseases. Moreover, though several dozen NF-κB inhibitors are under development, ~ 35% of them target Bruton tyrosine kinase, whose clinical indication is still blood cancer. Importantly, none of these NF-κB inhibitors target histone methylases, such as PRMT5.

It is worthwhile to note that there are other strategies to block NF-κB signaling, such as inhibiting NF-κB upstream activators like IKKβ^5^. The challenge with this approach is the reduced selectivity of IKKβ inhibitors given that they are often ATP-competitive and can bind to other kinases, causing undesirable effects. Blocking NF-κB nuclear translocation with JSH-23 reduces diabetic retinopathy^85^. However, this study was performed under the condition of hyperglycemia-induced NF-κB signaling, thereby limiting the extrapolation of the compound efficacy to other models. In our investigation, the observation of high expression of PRMT5 in both murine and human eye models suggests a novel mechanism for sustained NF-κB signaling in nvAMD.

In summary, we have identified a previously unreported role of PRMT5 in ocular angiogenesis. We employed a specific small molecule inhibitor of PRMT5 to inhibit PRMT5-mediated NF-κB activation and angiogenesis in vitro, and we compared this effect with PRMT5 knockdown in ocular endothelial cells. Hence, this study holds a valuable rationale to develop therapies targeting PRMT5-mediated NF-κB activation in treating not only nvAMD but also potentially other ocular neovascular diseases such as PDR and ROP. Future studies could include a thorough molecular investigation of cell-type specific PRMT5 expression and NF-κB regulation, and exploration of other potential transcriptional targets of inflammation and angiogenesis that may be regulated by PRMT5. On the therapeutic side, investigating the effects of PRMT5 inhibition in vivo holds promise to develop novel mono- or combination therapies targeting PRMT5 for treating ocular neovascular diseases.

## Supplementary Information

Supplementary Information.

## Data Availability

The datasets generated for this study can be obtained from the corresponding authors on reasonable request.
