# Supplementary material for: PRMT5 is a therapeutic target in choroidal neovascularization
Source: Sci Rep. 2023 Jan 31;13:1747. doi: 10.1038/s41598-023-28215-w (PMC9889383; doi:10.1038/s41598-023-28215-w)
Supplement: Supplementary file 1 — Supplementary Information. [file 41598_2023_28215_MOESM1_ESM.pdf]

Supplementary Information

PRMT5 is a therapeutic target in choroidal neovascularization

Anbukkarasi Muniyandi[1,$], Matthew Martin[2,$], Kamakshi Sishtla[1], Aishat Motolani[2], Mengyao Sun[2], Nathan R. Jensen[1], Xiaoping Qi[3], Michael E. Boulton[3], Lakshmi Prabhu[2], Tao Lu[2,4,5,*], Timothy W. Corson[1,2,4,*]

[1]Eugene and Marilyn Glick Eye Institute, Department of Ophthalmology, Indiana University School of Medicine, Indianapolis, IN 46202, USA

[2]Department of Pharmacology & Toxicology, Indiana University School of Medicine, Indianapolis, IN 46202, USA

[3]Department of Ophthalmology and Visual Sciences, University of Alabama at Birmingham, Birmingham, AL 35233, USA

[4]Department of Biochemistry & Molecular Biology, [5]Department of Medical & Molecular Genetics, Indiana University School of Medicine, Indianapolis, IN 46202, USA

[$]Equally contributed first authors

*Equal corresponding authors

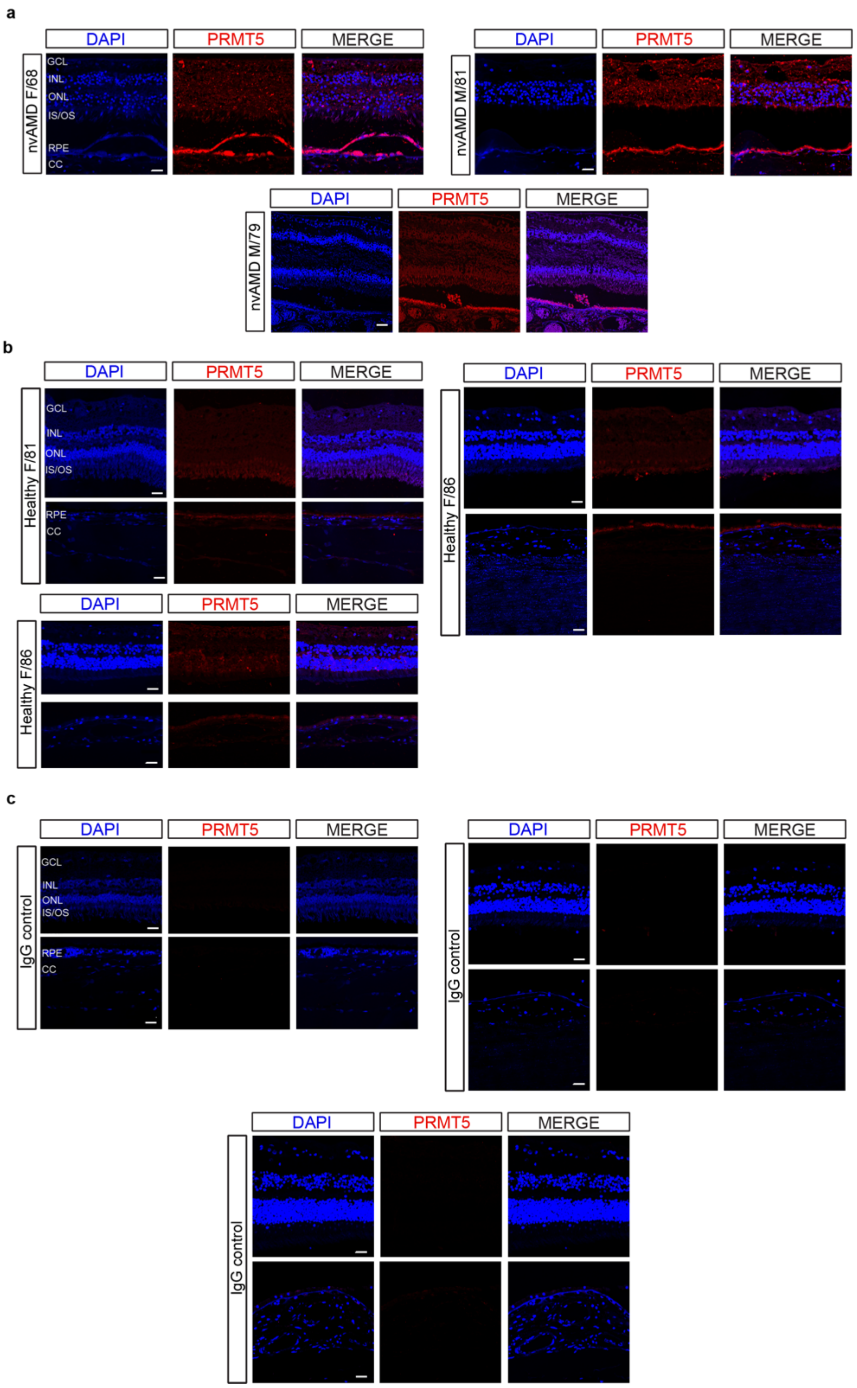

Supplementary Figure S1. PRMT5 is highly expressed in nvAMD eyes *vs.* healthy control eyes. PRMT5 immunostaining on sections of eyes from human (**a**) nvAMD patients (68 year old female, 81 year old male and 79 year old male) and (**b**) controls (81 year old female and two individual 86 year old females), (**c**) preimmune IgG control. DAPI (blue) shows the nuclei of the cells and red indicates PRMT5 expression in different layers of the retina, and in the RPE/choroid complex. Representative images shown from each of n=3 nvAMD patients and controls. Scale bars = 20 µm. GCL, ganglion cell layer; INL, inner nuclear layer; ONL, outer nuclear layer; IS/OS photoreceptor inner/outer segments; CC, choriocapillaris.

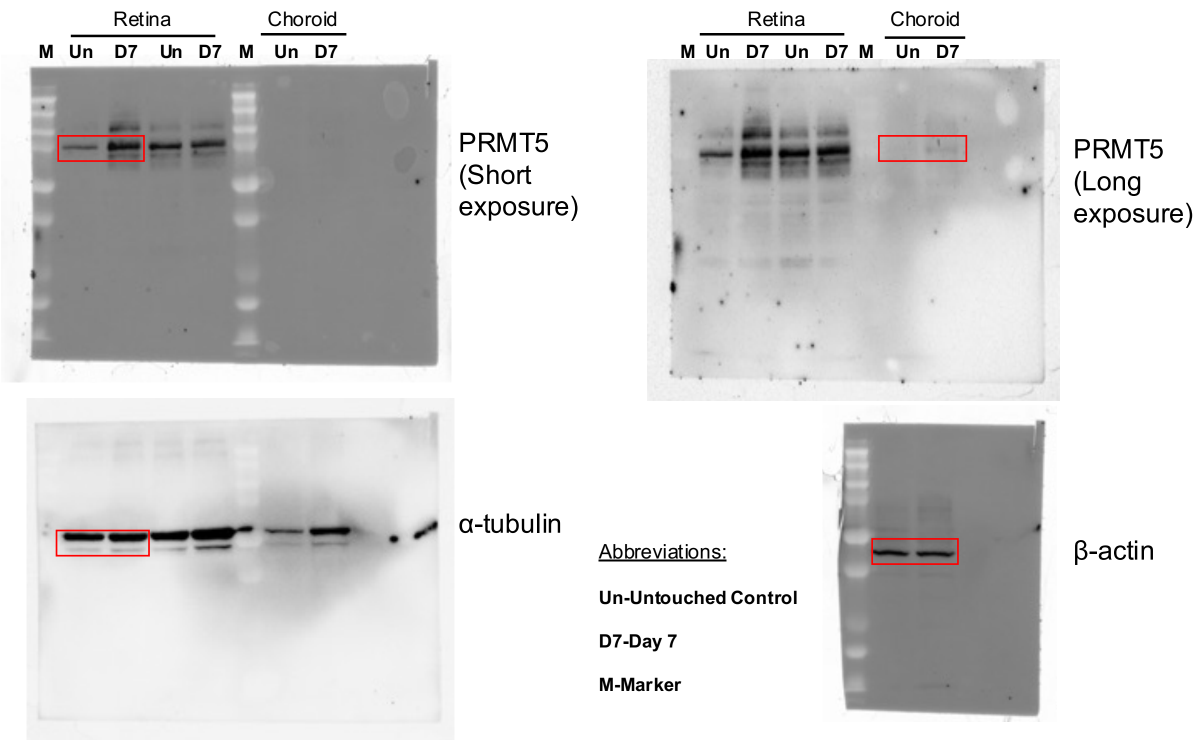

Supplementary Figure S2. Raw immunoblot data for images in Fig. 2b. The relevant part of each blot is shown in red boxes. Immunoblots anti-PRMT5 of indicated tissues were also reprobed for α-tubulin and cut and reprobed for β-actin (digital exposures).

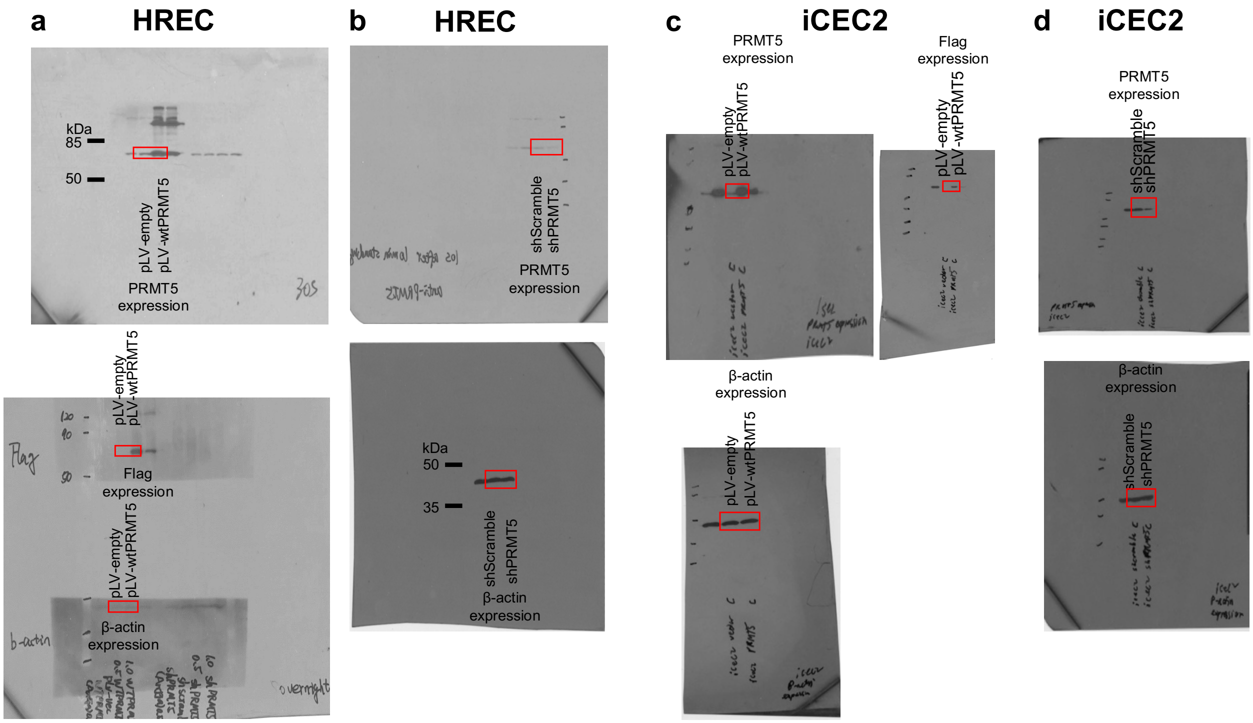

Supplementary Figure S3. Raw immunoblot data for images in Fig. 3a, b. The relevant part of each blot is shown in red boxes. (**a**) Immunoblot anti-PRMT5 of HREC with pLV-empty or pLV-wtPRMT5 (30 sec exposure). Blot was stripped and then cut horizontally at about 50 kDa. The top part was reprobed for total Flag (overnight exposure) and bottom part was reprobed with β-actin (overnight exposure) on the same film. (**b**) Immunoblot anti-PRMT5 of HRECs with shScramble or shPRMT5 (10 sec after 10 minute decay). Separately the same samples were re-run on a different blot and were probed for β-actin (30 sec exposure). (**c**) Immunoblot anti-PRMT5 of iCEC2 cells with pLV-empty or pLV-wtPRMT5 (1 sec exposure). Blot was stripped and reprobed for total Flag (30 sec exposure). Separately the same samples were re-run on a different blot and probed for β-actin (30 sec exposure). (**d**) Immunoblot anti-PRMT5 of iCEC2 cells with shScramble or shPRMT5 (20 sec exposure). Blot was stripped and reprobed for β-actin (30 sec exposure).

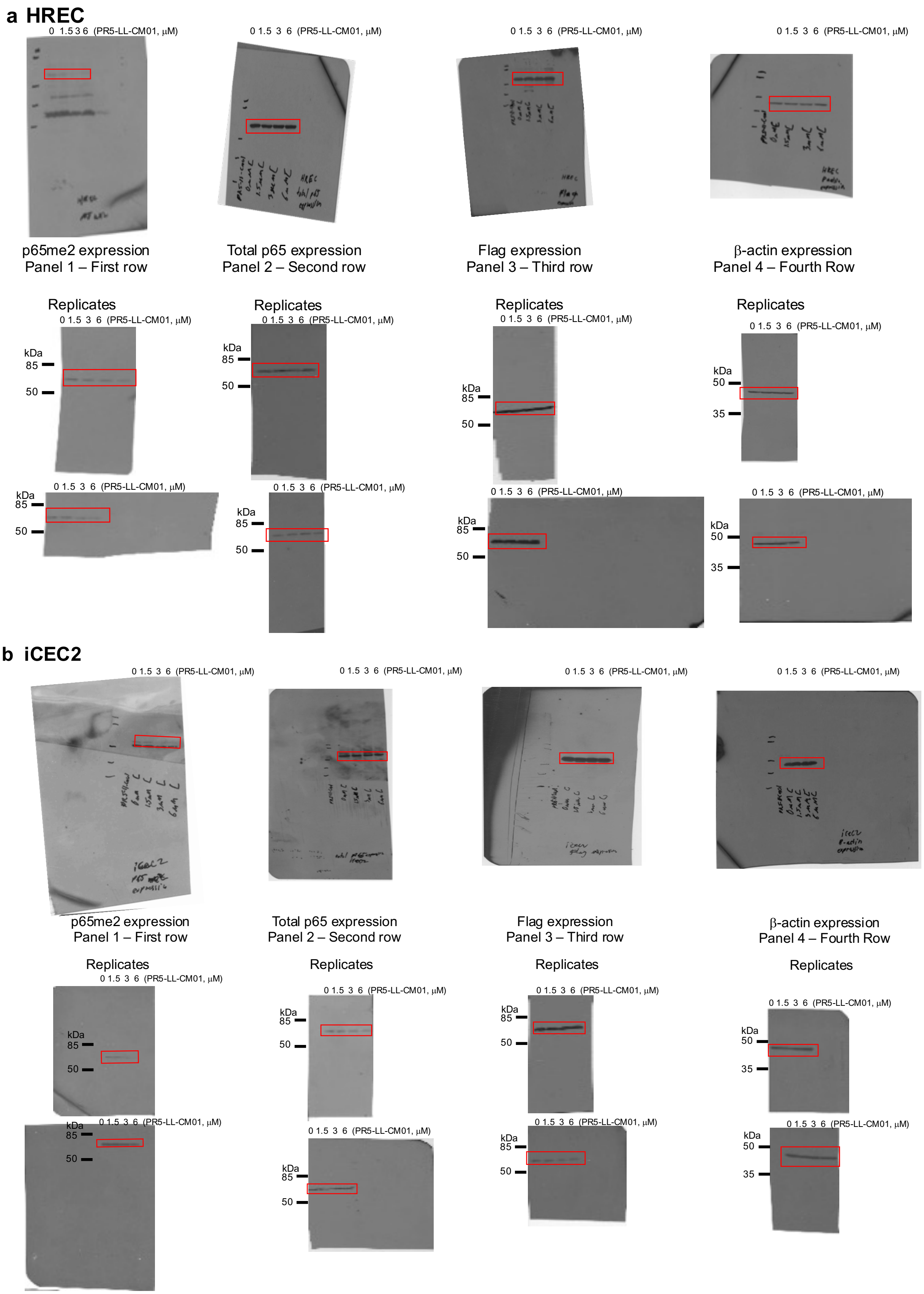
a HREC
0 1.5 3 6 (PR5-LL-CM01, µM)
p65me2 expression
Panel 1 – First row
Total p65 expression
Panel 2 – Second row
Flag expression
Panel 3 – Third row
β-actin expression
Panel 4 – Fourth Row
Replicates
kDa
85
50
35
b iCEC2
0 1.5 3 6 (PR5-LL-CM01, µM)
p65me2 expression
Panel 1 – First row
Total p65 expression
Panel 2 – Second row
Flag expression
Panel 3 – Third row
β-actin expression
Panel 4 – Fourth Row
Replicates
kDa
85
50
35

Supplementary Figure S4. Raw immunoblot data for images in Fig. 5a,b. The relevant part of each blot is shown in red boxes. (**a**) HRECs, (**b**) iCEC2. Immunoblot anti-p65me2 of indicated cells treated with PR5-LL-CM01 (0, 1.5, 3, 6 μM) (30 sec exposure) was stripped and reprobed for total p65 (30 sec exposure), stripped and reprobed for Flag (30 or 10 sec exposure), and stripped and reprobed for β-actin (10 sec exposure).

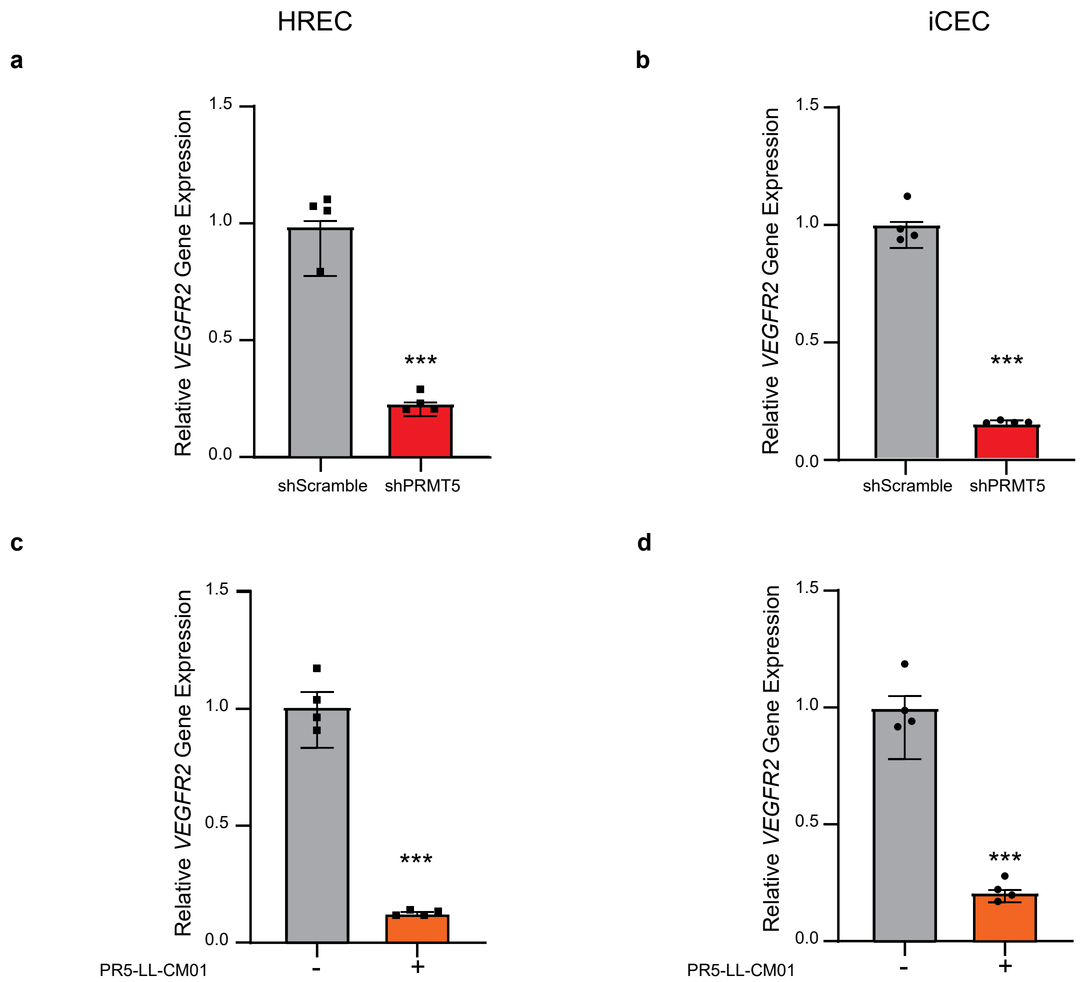

Supplementary Figure S5. PRMT5 is critical for *VEGFR2* expression in HRECs and iCEC2 cells. Quantitative PCR showing relative expression of *VEGFR2* transcript in (**a**, **c**) HRECs and (**b**, **d**) iCEC2 cells with (**a**, **b**) shScramble and shPRMT5 expression and (**c**, **d**) with or without 3 μM PR5-LL-CM01 treatment. *GAPDH* was used as an internal control and conditions were normalized to *GAPDH* expression. Mean±SD, n=4 replicates. ***$p<0.001$ *vs.* control, Student's t-test with Welch's correction.

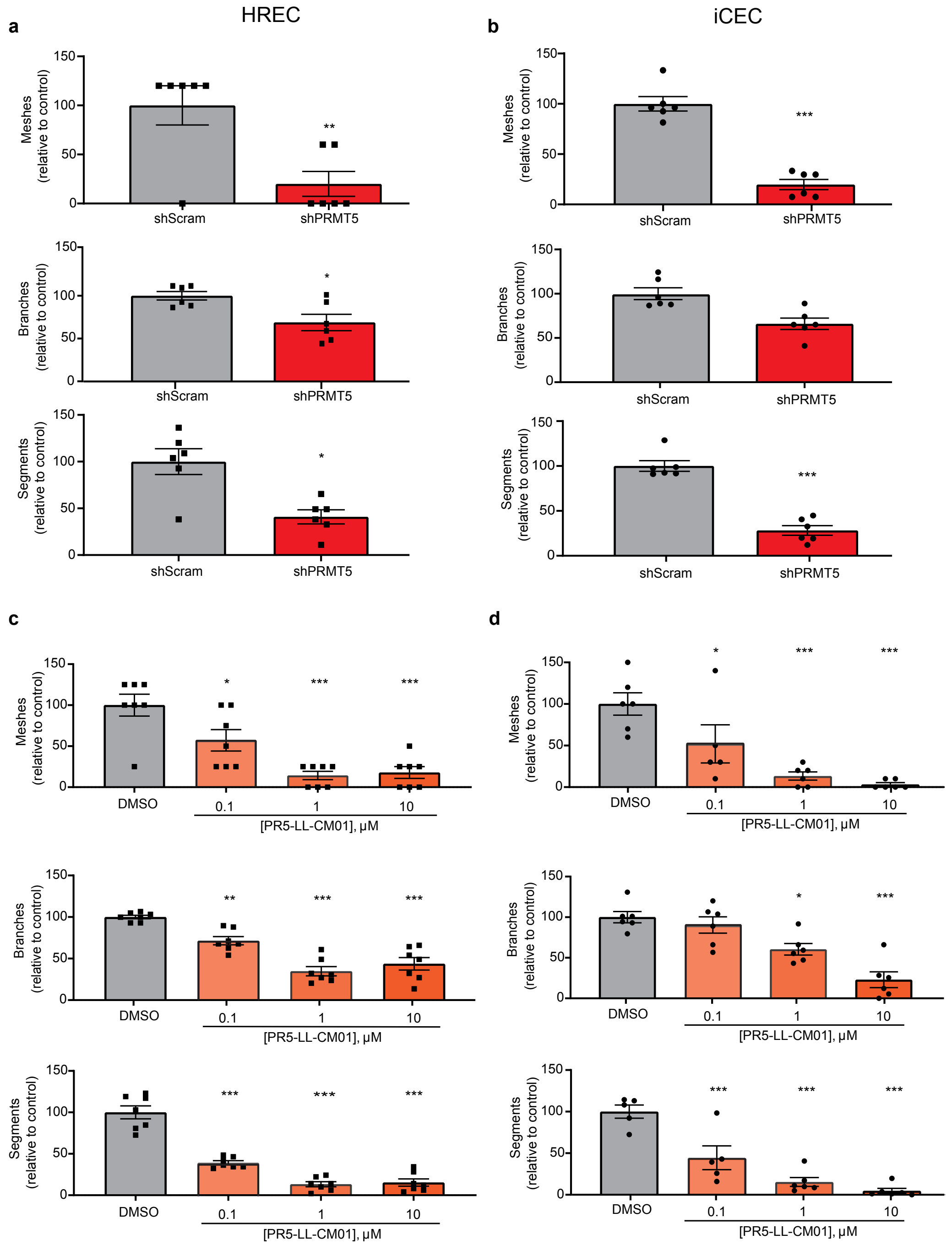

Supplementary Figure S6. shRNA knockdown-mediated inhibition of PRMT5 or treatment with PR5-LL-CM01 reduces other measures of tube formation in HRECs and iCEC2 cells**.** Quantification of tube formation using number of meshes, branches and segments in (**a**) HRECs or (**b**) iCEC2 cells transduced with shScramble vector or shPRMT5, (**c**) HRECs or (**d**) iCEC2 cells exposed to PR5-LL-CM01, showing that knockdown of PRMT5 or PR5-LL-CM01 treatment diminishes the parameters of number of meshes, branches and segments in comparison to shScramble/DMSO controls. Mean±SEM, n=6-12 images. *p<0.05; **p<0.01; ***p<0.001 *vs.* control, one-way ANOVA with Dunnett's post hoc test. Representative data from three biological replicates.

Supplementary Table S1. Antibodies and lectin used in the study.

Antibody	Supplier	Catalogue #	Purpose and dilution
Biotin-conjugated, *Griffonia simplicifolia* Isolectin B4 (GS-IB4)	Invitrogen, Waltham, MA, USA	121414	Flat-mount staining (1:250), Mouse immunostaining (1:250)
Rabbit anti-PRMT5	Abcam, Waltham, MA, USA	ab109451	Flat-mount staining (1:150), Immunostaining (human sections: 1:100 and mouse sections: 1:150), Immunoblotting for tissues (1:1000), Immunoblotting for cells (1:3000)
Alexafluor 555-conjugated goat anti-rabbit antibody	Invitrogen, Waltham, MA, USA	A21428	Flat-mount staining (1:250), Immunostaining (human sections: 1:200, mouse sections: 1:250)
Rabbit IgG control	R&D systems, Minneapolis, MN, USA	AB-105-C	Immunostaining (human sections: 1:100, mouse sections: 1:150)
β-actin (mouse)	Sigma-Aldrich, St. Louis, MO, USA	A5316	Immunoblotting for tissues (1:1000), Immunoblotting for cells (1:5000)
Anti-rabbit IgG peroxidase conjugated	Rockland, Rockland, MD, USA	611-1302	Immunoblotting for tissues (1:10,000)
Anti-mouse IgG peroxidase conjugated	Rockland, Rockland, MD, USA	610-1302	Immunoblotting for tissues (1:10,000)
Rabbit anti-p65	Santa Cruz Biotech, Dallas, TX, USA	sc109	Immunoblotting for cells (1:3000)
Rabbit anti-p65me2	Genscript, Piscataway, NJ, USA	custom antibody	Immunoblotting for cells (1:750)
Mouse anti-FLAG M2	Millipore-Sigma, St. Louis, MO, USA	F-1804	Immunoblotting for cells (1:3000)
Goat anti-rabbit IgG (H+L) secondary antibody, HRP	ThermoFisher, Waltham, MA, USA	31460	Immunoblotting for cells (1:3000)
Goat anti-mouse IgG (H+L) secondary antibody, HRP	ThermoFisher, Waltham, MA, USA	62-6520	Immunoblotting for cells (1:3000)

Supplementary Table S2. Primers used for qPCR.

Primer Name	Primer Sequence
GAPDH-F	CCATCACCATCTTCCAGGAGCG
GAPDH-R	AGAGATGATGACCCTTTTGGC
VEGFA-F	TTGCCTTGCTGCTCTACCTCCA
VEGFA-R	GATGGCAGTAGCTGCGCTGATA
TNFA-F	TGGCCCAGGCAGTCAGA
TNFA-R	GGTTTGCTACAACATGGGCTACA
VEGFR2-F	CCGGCCTGTGAGTGTAAAAAC
VEGFR2-R	CGTCTGGTTGTCATCTGGGA
